# *NOTCH1*-mutated chronic lymphocytic leukemia displays high endoplasmic reticulum stress response with druggable potential

**DOI:** 10.3389/fonc.2023.1218989

**Published:** 2023-09-25

**Authors:** Estevão Carlos Silva Barcelos, Chiara Rompietti, Francesco Maria Adamo, Erica Dorillo, Filomena De Falco, Beatrice Del Papa, Stefano Baldoni, Manuel Nogarotto, Angela Esposito, Silvia Capoccia, Clelia Geraci, Daniele Sorcini, Arianna Stella, Roberta Arcaleni, Valentina Tini, Flávia Imbroisi Valle Errera, Emanuela Rosati, Paolo Sportoletti

**Affiliations:** ^1^ Department of Medicine and Surgery, Institute of Hematology, Centro di Ricerca Emato-Oncologica (CREO), University of Perugia, Perugia, Italy; ^2^ Postgraduate Program in Biotechnology, Federal University of Espírito Santo, Vitória, Brazil; ^3^ Department of Medicine and Sciences of Aging, “G. d’Annunzio” University of Chieti-Pescara, Chieti, Italy; ^4^ Department of Medicine and Surgery, Biosciences and Medical Embryology Section, University of Perugia, Perugia, Italy

**Keywords:** chronic lymphocytic leukemia, NOTCH1 mutation, unfolded and integrated stress response, endoplasmic reticulum stress (ER stress), curcumin

## Abstract

**Introduction:**

Constitutive activation of *NOTCH1*-wild-type (NT1-WT) signaling is associated with poor outcomes in chronic lymphocytic leukemia (CLL), and *NOTCH1* mutation (c.7541_7542delCT), which potentiates NOTCH1 signaling, worsens the prognosis. However, the specific mechanisms of NOTCH1 deregulation are still poorly understood. Accumulative evidence mentioned endoplasmic reticulum (ER) stress/unfolded protein response (UPR) as a key targetable pathway in CLL. In this study, we investigated the impact of NOTCH1 deregulation on CLL cell response to ER stress induction, with the aim of identifying new therapeutic opportunities for CLL.

**Methods:**

We performed a bioinformatics analysis of *NOTCH1*-mutated (NT1-M) and NT1-WT CLL to identify differentially expressed genes (DEGs) using the rank product test. Quantitative real-time polymerase chain reaction (qPCR), Western blotting, cytosolic Ca^2+^, and annexin V/propidium iodide (PI) assay were used to detect curcumin ER stress induction effects. A median-effect equation was used for drug combination tests. The experimental mouse model Eμ-TCL1 was used to evaluate the impact of ER stress exacerbation by curcumin treatment on the progression of leukemic cells and NOTCH1 signaling.

**Results and discussion:**

Bioinformatics analysis revealed gene enrichment of the components of the ER stress/UPR pathway in NT1-M compared to those in NT1-WT CLL. Ectopic expression of *NOTCH1* mutation upregulated the levels of ER stress response markers in the PGA1 CLL cell line. Primary NT1-M CLL was more sensitive to curcumin as documented by a significant perturbation in Ca^2+^ homeostasis and higher expression of ER stress/UPR markers compared to NT1-WT cells. It was also accompanied by a significantly higher apoptotic response mediated by C/EBP homologous protein (CHOP) expression, caspase 4 cleavage, and downregulation of NOTCH1 signaling in NT1-M CLL cells. Curcumin potentiated the apoptotic effect of venetoclax in NT1-M CLL cells. In Eμ-TCL1 leukemic mice, the administration of curcumin activated ER stress in splenic B cells *ex vivo* and significantly reduced the percentage of CD19^+^/CD5^+^ cells infiltrating the spleen, liver, and bone marrow (BM). These cellular effects were associated with reduced NOTCH1 activity in leukemic cells and resulted in prolonged survival of curcumin-treated mice. Overall, our results indicate that ER stress induction in NT1-M CLL might represent a new therapeutic opportunity for these high-risk CLL patients and improve the therapeutic effect of drugs currently used in CLL.

## Introduction

Accumulating evidence indicates a key role of deregulated NOTCH1 signaling in chronic lymphocytic leukemia (CLL). We and others reported a high frequency of *NOTCH1* mutations (1, 2) affecting the clinical outcome of patients with CLL. Mutations in the *NOTCH1* gene, most commonly as a 2-bp frameshift deletion (c.7541_7542delCT), are implicated in constitutive activation of NOTCH1 signaling and distinct transcriptional profile in CLL (3–5).

CLL cells were shown to require activation of the endoplasmic reticulum (ER) stress response for survival (6). In stressed cells, the ER triggers a cascade of signaling known as the unfolded protein response (UPR), caused by the accumulation of unfolded or misfolded proteins in the ER. UPR is mediated by three ER transmembrane sensors, protein kinase RNA-like endoplasmic reticulum kinase (PERK), inositol-requiring enzyme 1 (IRE1), and activating transcription factor 6 (ATF6), all activated by the removal of the chaperone glucose-regulated protein 78 (GRP78/*HSPA5*, also known as BiP). Then, as part of UPR compensatory mechanisms, BiP translocates from the ER membrane to the ER lumen, where it associates with unfolded proteins, allowing protein homeostasis restoration and cell survival maintenance (7). Harding et al. (8) also found PERK as an essential translational regulator during UPR, with its activating downstream factor ATF4 appearing to be indispensable to controlling transcriptional programs and cell survival (9). However, under severe and prolonged stress, this ER homeostatic program can drive signaling toward cell death through interactions with the pro-apoptotic C/EBP homologous protein (CHOP) and suppression of anti-apoptotic members of the BCL2 family (10, 11). Other compensatory mechanisms include IRE1 and ATF6 sensors. Upon BiP release, IRE1 induces the splicing of XBP1 messenger RNA (mRNA), which, once translated into the active sXBP1, promotes the expression of components related to protein folding, ER-associated degradation (ERAD), and protein quality control (12). Another branch of UPR is mediated by ATF6, which, upon ER stress, is transferred to the Golgi apparatus and is cleaved by membrane-bound site-1 (S1P) and site-2 (S2P) proteases into an active form, which induces the expression of chaperones and UPR components (7).

Our previous results demonstrated that severe ER stress-induced apoptosis was amplified by NOTCH1 suppression in CLL cells, suggesting a role of the NOTCH1 pathway in regulating adaptive or apoptotic responses in ER stress conditions (13). However, how NOTCH1 deregulation influences CLL cell response to ER stress induction remains to be investigated, raising the possibility of identifying new therapeutic opportunities for CLL patients. In this study, we report a set of genes involved in response to stress, including ER stress, which are differentially expressed in *NOTCH1*-mutated (NT1-M) CLL compared to *NOTCH1*-wild-type (NT1-WT) CLL. The induction of ER stress downregulates NOTCH1 signaling in NT1-M CLL cells *in vitro* and exerts anti-leukemic activity in Eµ-TCL1 mice, a CLL model with aberrantly active ER response (14). Overall, our results suggest that ER stress induction might open a new perspective on the therapeutic approach for these high-risk CLL patients.

## Materials and methods

### Data source and bioinformatics analysis

The data analyzed were extracted from National Center for Biotechnology Information (NCBI) Gene Expression Omnibus (GEO) datasets GSE75122 (15), GSE137024 (16), and GSE92626 (4) (**Supplementary Table S1**). To identify differentially expressed genes (DEGs), data were processed as described elsewhere (17, 18) (**Supplementary Figure S1**). We used the R packages SVA (ComBat function) (19) to adjust combined data for batch effects and RankProd (20) to calculate the rank product (RP), a non-parametric method based on the estimated percentage of false predictions (pfp). Gene Ontology and Reactome pathway analyses were performed using gprofiler (21) and the Reactome database (22), respectively, with a threshold of 0.05 (both). To visualize significantly enriched Reactome pathways [false discovery rate (FDR)< 10%], “makeDendrogram.py” (pyEnrichment: https://github.com/ofedrigo/pyEnrichment) and custom in-house R scripts were used to construct bubble plots using multidimensional scaling to calculate an optimal two-dimensional (2D) arrangement of pathways based on a distance matrix of between-pathway semantic scores. All these analyses were carried out in the R environment. The gene–gene interaction was evaluated with the GeneMANIA tool (23) using Cytoscape v3.9.1.

### Primary CLL cells

All participants signed written informed consent forms in accordance with the Declaration of Helsinki, and laboratory protocols were approved by the Institutional Review Board of the University of Perugia. Primary CLL cells were isolated from peripheral blood (PB) with a purity of 93.8% ± 2.7% CD19^+^/CD5^+^, assessed by flow cytometry using anti-human CD45, CD19, CD5, CD11b, and CD3 monoclonal antibodies on 7AAD-negative cells (**Supplementary Table S2**), and characterized for IGHV and SF3B1 mutational status and the main cytogenetic abnormalities as described elsewhere (24–26). The *NOTCH1* mutation (c.7541_7542delCT) allelic burden of CLL cells was determined by digital PCR (ddPCR), as previously described (27), and stratified according to allele mutation frequency as low (from 0.03% to 12%) and high (above 12%), as described elsewhere (5, 28).

### Cell culture

Cells were cultured at a density of 2 × 10^6^ cells/ml in complete medium, consisting of RPMI 1640 supplemented with 10% heat-inactivated fetal bovine serum (Gibco), 2 nM L-glutamine, 100 U/ml penicillin, and 100 μg/ml streptomycin (all from Invitrogen), with the following agents: dimethyl sulfoxide (DMSO) as a vehicle, curcumin (Sigma-Aldrich, St. Louis, MO, USA), and/or ABT-199/venetoclax (Selleck Chemicals, Houston, TX, USA). EDTA (0.5 mM) was added to specifically activate NOTCH1 signaling.

### Transduction of PGA1 cells

Lentiviral packaging was conducted using 293T cells at a confluency of 70%–80%. 293T cells (15 × 10^4^ cells) were transiently transfected with an FG12-based lentiviral vector expressing green fluorescent protein (GFP), containing pNICD WT or pNICD-mutated fragment (c.7541_7542delCT), or is empty, used as a mock control (7.5–10 µg plasmid DNA). Lentiviral vector stocks were generated using pCMVR8.74 (packaging plasmid) and pMD2.G (VSV-G envelope expressing plasmid). All plasmids were obtained from Addgene (Watertown, Massachusetts). FG12 was a gift from David Baltimore (Addgene plasmid #14884) (29), and pCMVR8.74 and pMD2.G were a gift from Didier Trono (Addgene plasmids #22036 and #12259, respectively).

The transfection was performed using T-Pro NTR II transfection reagent (T-Pro Biotechnology) in Opti-MEM medium (Gibco, Thermo Fisher). After collection and filtration of the viral supernatant, all PGA1 cells were subjected to static transduction for 48 h. The transduction was carried out at a multiplicity of infection (MOI) of 20, with the addition of 6 μg/ml polybrene and the transduction enhancer surfactant Synperonic F108 (Sigma-Aldrich).

After fluorescence-activated cell sorting (FACS), the percentage of GFP-positive cells evaluated at flow cytometry was 93.15% for mock cells, 90.68% for NICD WT cells, and 89.89% for NICD-mutated cells (**Supplementary Figure S2A**). Western blotting and ddPCR were used to determine the NICD protein levels and *NOTCH1* mutational status, respectively, of transduced PGA1 cells compared to those of untransduced PGA1 (**Supplementary Figures S2B, C**).

### Quantitative real-time PCR

RNA was extracted using the RNeasy kit (Qiagen, Hilden, Germany), as per the manufacturer’s protocol, and complementary DNA (cDNA) synthesis was performed using an RT reagent kit (Takara Biotechnology Co., Ltd.). Quantitative real-time PCR (qPCR) was carried out using the 7300HT fast real-time PCR system (Applied Biosystems, Warrington, UK) and Power SYBR Green PCR master mix (Applied Biosystems, Warrington, UK). The relative mRNA expression levels were calculated using the 2^−ΔΔCq^ method and normalized to the internal control gene GAPDH. The sequences of primers are shown in **Supplementary Table S3**.

### Western blot assay

Whole-cell lysates were extracted in a cold radioimmunoprecipitation assay (RIPA) lysis buffer containing a protease/phosphatase inhibitor cocktail (Sigma‐Aldrich). The protein concentration was determined by Bradford assay, and proteins were fractionated by sodium dodecyl sulfate–polyacrylamide gel electrophoresis (SDS-PAGE) and electro-transferred onto a nitrocellulose membrane (Millipore, MA, USA). Blots were blocked with 5% dried non-fat milk powder in Tris-buffered saline Tween 20 (TBST) and incubated overnight at 4°C against the primary antibodies listed in **Supplementary Table S2**. After washing, membranes were incubated with horseradish peroxidase-conjugated secondary antibodies (Cell Signaling Technology, Beverly, MA) for 1 h and signals were obtained on a transilluminator (ChemiDoc™ MP imaging system, Bio-Rad Lab., Milan, Italy), and densitometric quantification was performed using the ImageLab software (Bio-Rad Lab.).

### Fluo-4 calcium assay

Cytosolic Ca^2+^ fluxes were determined by using the calcium-sensitive dye Fluo-4 Direct™ (Thermo Scientific), according to the manufacturer’s protocol. In brief, CLL primary cells were washed once to remove the medium. The cell pellet was then resuspended in Fluo-4 Direct™ calcium assay buffer at a density of approximately 2.5 × 10^6^ cells/ml. The plate was incubated at 37°C and 5% CO_2_ for 60 min to allow the cells to settle. Subsequently, an equal volume of Fluo-4 Direct calcium reagent solution, containing 5 mM probenecid, was added to the cells, which were further incubated for an additional 45 min. Samples were acquired for 60 s to determine the basal levels of Ca^2+^. Then, either calcium ionophores (2 μM; A23187, Sigma-Aldrich), curcumin (15 μM), or DMSO (0.05%) was added to the cells, and data acquisition continued for an additional 420 s.

The acquisition was performed using a FACSCanto flow cytometer (BD Biosciences). The fluorescence intensity at 516 nm was monitored after excitation at 494 nm. The Ca^2+^ concentration was calculated, as described elsewhere (30), using the equation


$$\left[\text{calcium }\left(\text{nmol/L}\right)\right]\;={\text{ K}}_{\text{d}}\times (\text{R}-{\text{R}}_{\text{min}}/({\text{R}}_{\text{max}}-\text{R})$$

where K_d_ represents the dissociation constant of calcium bound to the fluorochrome (as provided by the kit datasheet), R is the peak fluorescence observed in response to curcumin or the fluorescence at the basal level, R_min_ is the fluorescence measured at the end of acquisition (480 s), and R_max_ is the peak of fluorescence in response to ionophore.

### Analysis of cell apoptosis and drug combination assay

Cell apoptosis was evaluated by flow cytometry after annexin V/propidium iodide (AnV/PI) double staining performed with a commercial kit (Immunotech, Beckman Coulter), according to the manufacturer’s instructions. Results were analyzed by FlowJo software version 10 (FlowJo, LLC, Ashland, OR, USA). For drug combination studies, CLL cells were incubated with different concentrations of curcumin (7.5, 15, and 30 µM) and venetoclax (1, 2, and 4 nM) alone and in combination. To calculate the combination index (CI) and the dose reduction index (DRI), data from AnV/PI assay were analyzed by using the CompuSyn software (ComboSyn Inc., Paramus, NJ, USA) (31). Based on the Chou–Talalay method, CompuSyn considers the complete shape of the growth inhibition curve. The CI was calculated using the formula CI = (D)1/(Dx)1 + (D)2/(Dx)2. (D)1 and (D)2 represent the doses of drug 1 and drug 2 in combination, and (Dx)1 and (Dx)2 represent the doses of drug 1 and drug 2 alone that produce the same effect level as the combination. A CI value less than 1 indicates synergism (greater than additive effect), a CI value equal to 1 suggests additivity (additive effect), and a CI value greater than 1 indicates antagonism (less than additive effect). The drug combination analysis of venetoclax and curcumin was conducted using a constant ratio of 1:7.5. *In vivo* experiments C57BL/6 (WT) and Eµ-TCL1 mice [provided by Prof. Paolo Ghia and generated by Prof. Carlo Croce (32)] were kept in specific pathogen-free conditions, and experiments were carried out in accordance with the protocols approved by the Italian Health Ministry (authorization no. 1155/2015-PR and no. 971/2020-PR). Eµ-TCL1 mice with 5% ± 3.25% of CD19^+^/CD5^+^ cells in PB received curcumin (50 mg/kg/day) or corn oil (vehicle) (Sigma-Aldrich), both administered by intraperitoneal injection for 2 months once daily. PB was analyzed by bi-monthly bleedings and at sacrifice. Bone marrow (BM), spleens, and livers were also processed and analyzed. BM and splenic cells were stained with anti-mouse CD19 and CD5 (**Supplementary Table S2**) for cell sorting, performed using BD FACSAria™ III, considering 95% purity. All panel gates were drowned to exclude non-viable cells and debris.

### Statistical analysis

Statistical analyses were performed using GraphPad Prism (GraphPad Software Inc., La Jolla, CA, USA). Statistical differences between mean values were evaluated using Student’s t-test for parametric data. Non-parametric data were analyzed by Wilcoxon (paired data) or Mann–Whitney (non-paired data) test. A one-way ANOVA test was used for the three groups’ comparison. Kaplan–Meier survival curves were compared using the Mantel–Cox rank sum test. Results were considered significant with a *P*-value< 0.05.

## Results

### *NOTCH1*-mutated CLL cells displayed an altered ER stress response/ISR/UPR signature

**Figure 1:**
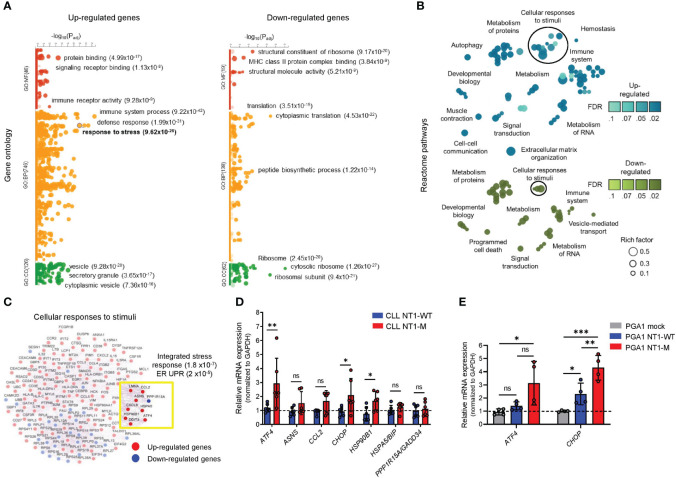
ER UPR markers are enriched in *NOTCH1*-mutated CLL cells. **(A)** Gene Ontology enrichment analysis of up- (left panel) and downregulated DEGs (right panel) in NT1-M versus NT1-WT CLL samples, with three of the most significant pathways for MF, BP, and CC shown next to the dots with their *P*-values. “Response to stress” is represented by the highlighted circle, and its *P*-value is shown in bold. MF: molecular function; BP: biological process; CC: cellular component. **(B)** The bubble plot show enriched Reactome pathways for upregulated DEGs (blue, upper panel) and downregulated DEGs (green, bottom panel). For each pathway, the false discovery rate (FDR) is represented by shading, and the rich factor (ratio of the number of DEGs annotated in a pathway to the number of all genes annotated in this pathway) is represented by bubble size. Groups of related pathways are labeled with a general descriptive term as opposed to individual pathway names. **(C)** The gene interaction network of genes involved in “cellular responses to stimuli” shows the co-expression (represented by purple edges) among up- (red nodes) and downregulated (blue nodes) DEGs. Genes highlighted in the yellow box are enriched in the integrated stress response (1.8 × 10^−7^) and ER UPR (2 × 10^−5^). **(D)** Bar graphs with data points show the gene expression of ER/UPR markers, assessed by qPCR, in NT1-M CLL primary cells (N = 6) compared with NT1-WT (N = 6). mRNA levels were normalized to GAPDH and are represented as fold change using NT1-WT cells as a reference set to 1 (dashed line). Data are presented as mean ± SD. *P< 0.05, **P< 0.01; ns, not significant as determined by the Mann–Whitney unpaired test. **(E)** Bar graphs with data points show the gene expression of the ER/UPR markers *ATF4* and *CHOP* in PGA1 cells transduced with NICD-mutated and NICD WT fragment of four independent tests. mRNA levels were normalized to GAPDH and are represented as fold change by using PGA1 with an empty vector (mock) as a reference set to 1 (dashed line). Data are presented as mean ± SD. *P< 0.05, **P< 0.01, ***P< 0.001; ns, not significant as determined by using one-way ANOVA (Geisser–Greenhouse correction).

We first performed a bioinformatics analysis of NT1-M and NT1-WT CLL (21 vs. 31 samples, respectively) to identify DEGs, using three independent gene expression datasets (GSE75122, GSE137024, GSE92626). We found 620 DEGs in the comparison between NT1-M and NT1-WT CLL cells (470 upregulated; 150 downregulated; **Supplementary Table S4**). These DEGs were enriched for distinct Gene Ontology (GO) categories (**Supplementary Table S5**), and among the increased set of biological processes, *NOTCH1* mutation drove enrichment of the “immune system process” (*P* = 9.22 × 10^−42^), “defense response” (*P* = 1.99 × 10^−31^), and “response to stress” (*P* = 9.62 × 10^−28^) (**Figure 1A**). The downregulated categories evidenced enrichment for “cytoplasmic translation” (*P* = 4.53 × 10^−22^), “translation” (*P* = 3.51 × 10^−15^), and the “peptide biosynthetic process” (*P* = 1.22 × 10^−14^). To identify altered canonical pathways, we performed an overrepresentation and topology of DEGs with the Reactome tool, which revealed a greater number of terms related to cellular responses to stimuli (circled in **Figure 1B**), including “PERK regulates gene expression” and “ATF4 activates genes in response to ER stress” (**Supplementary Table S6**). Genes involved in these pathways were further evaluated in a gene–gene interaction approach. Enrichment analysis revealed deregulation of the integrated stress response (ISR) (*P* = 1.8 × 10^−7^) and UPR (*P* = 2 × 10^−5^) genes in NT1-M CLL (**Figure 1C**).

Analysis of these genes in our CLL cells (**Supplementary Table S7**) showed that *ATF4*, *CHOP*, and *HSP90B1* mRNA expression was higher in NT1-M CLL compared to that in NT1-WT CLL (*P* = 0.008, *P* = 0.041, and *P* = 0.026, respectively) (**Figure 1D**).

In an attempt to better define the association of NT1 mutation with ER stress, we transduced the PGA1 CLL cell line with NICD WT or NICD-mutated fragment or an empty vector (mock). As shown in **Supplementary Figure S2A**, the percentages of PGA1 cells transduced with NICD WT or NICD-mutated fragment were 90.68 and 89.89, respectively. Western blot (WB) analysis of NICD showed that i) mock PGA1 cells express NICD WT levels similar to those of the untreated PGA1 control; ii) cells transduced with NICD WT overexpress NICD WT compared to mock cells; and iii) cells transduced with NICD-mutated fragment express NICD mutation, in addition to NICD WT (**Supplementary Figure S2B**). Using ddPCR multiplex assay, we confirmed NICD-mutated frequency in PGA1 transduced cells with *NOCTH1* mutation (**Supplementary Figure S2C**). When we analyzed the effect of NICD transduction on ER stress markers, we found that PGA1 cells overexpressing NICD mutation showed increased expression of *ATF4* and *CHOP* mRNA compared to both PGA1 mock (*P* = 0.021 and *P* = 0.0002, respectively) and PGA1 cells transduced with NICD WT (*P* = 0.06 and *P* = 0.0064, respectively), as shown in **Figure 1E**. Altogether, these data suggested that *NOTCH1* mutation are associated with ISR/UPR/ER stress response markers in CLL.

### *NOTCH1*-mutated CLL cells were sensitive to ER stress induced by curcumin

Based on the above evidence that *NOTCH1* mutation alters the expression of specific ER/UPR markers in CLL, we investigated whether NOTCH1 deregulation influenced CLL cell response to ER stress induction, by using curcumin, a natural ER stress inducer (33–36).

**Figure 2:**
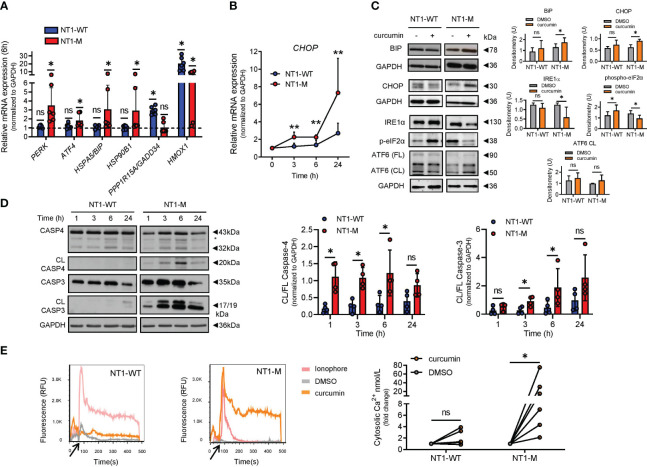
UPR/ER stress response differs between NT1-M and NT1-WT CLL cells after curcumin treatment. Primary CLL cells (2 × 10^6^ cells/ml) were incubated with 15 µM curcumin or vehicle DMSO at 0.05%. **(A)** Bar graphs showing the transcriptional expression of ER UPR markers *PERK*, *ATF4*, *BIP/HSPA5*, and *HSP90B1* and the stress-overcoming genes *GADD34* and *HMOX1*, in NT1-WT and NT1-M cells assessed via qPCR (N = 6, per group), after 6 h of incubation. mRNA levels were normalized to GAPDH and are represented as fold change using cells incubated with DMSO as a reference set to 1 (dashed line). **(B)** Kinetics of mRNA expression of the pro-apoptotic transcription factor *CHOP* after curcumin treatment (N = 8, per group). **(A, B)** Data are presented as mean ± SD. *P< 0.05; ns, not significant as determined by Wilcoxon paired test **(A)**. **P< 0.01 as determined by Mann–Whitney unpaired test. **(C)** Representative Western blots (left) of BiP, CHOP, IRE1α, phosphorylated eIF2α (p-eIF2α), and full-length (FL) and cleaved (CL) ATF6 after 24-h treatment with curcumin or DMSO and bar graphs (right) with densitometry data of NT1-WT and NT1-M cells (N = 6, per group). **(D)** Representative Western blots (left) of caspase 4 and caspase 3 cleavage at the indicated time points and bar graphs (right) with data points of densitometric analysis of CL/FL caspase 4 and CL/FL caspase 3 in NT1-WT and NT1-M cells (N = 4, per group). Data are presented as mean ± SD. *P< 0.05; ns, not significant as determined by Mann–Whitney unpaired test. **(E)** Cytosolic Ca^2+^ measurement of CLL cells loaded with Fluo-4. Left, relative fluorescence units (RFU) from Fluo-4 were recorded during a 60-s period in basal conditions (black arrow) and after the addition of calcium ionophores (2 μM), DMSO, or curcumin, for 420 s. Right, dot-and-line diagram of the fold change of cytosolic Ca^2+^ levels (nmol/L) using cells incubated with DMSO as a reference set to 1, in NT1-WT or NT1-M (N = 6, per group). *P< 0.05; ns, not significant as determined by Wilcoxon paired test.

Curcumin-treated NT1-M cells expressed increased levels of *PERK* (*P* = 0.031), *ATF4* (*P* = 0.031), *BIP* (*P* = 0.031), and *HSP90B1* (*P* = 0.031) mRNAs compared to DMSO-treated cells, whereas *GADD34* and *HMOX1* mRNAs, essential in overcoming ER stress, were upregulated in NT1-WT cells (*P* = 0.031, both) (**Figure 2A**). After curcumin treatment, even *CHOP* mRNA was increased in NT1-M compared to NT1-WT cells (**Figure 2B**). Analysis of BiP/HSPA5 and CHOP proteins showed that curcumin significantly increased their levels in NT1-M cells compared to DMSO (**Figure 2C**). In NT1-WT, the levels of both proteins were not significantly changed, suggesting that in the absence of *NOTCH1* mutation, the ER stress induced by curcumin is milder. We also examined the effect of curcumin on some homeostatic UPR markers. We analyzed i) the expression of IRE1α and ATF6 proteins, because either IRE1-mediated activation of XBP1 or activation of ATF6 induces the expression of chaperones and UPR components important for counteracting ER stress and restoring ER homeostasis (7, 12), and ii) the phosphorylation of eIF2α, a target of the PERK pathway, which leads to global inhibition of protein translation (8). **Figure 2C** shows that IRE1α levels were not affected by curcumin in NT1-WT CLL cells compared to controls, while they were reduced in NT1-M cells. eIF2α phosphorylation was increased by curcumin in CLL NT1-WT cells compared to that in controls, while it was reduced in NT1-M cells. Levels of ATF6, both full-length (FL) and the active cleaved (CL) fragment, were unaffected by curcumin either in NT1-WT or NT1-M CLL. Altogether, these results indicate that *NOTCH1* mutation is associated with an exacerbated ER stress response after stimulation with an ER stress inducer, as also supported by experiments with thapsigargin (**Supplementary Figure S3**).

Massive ER stress can trigger apoptosis via caspase 4 activation (13). When we analyzed the effect of curcumin on caspase 4 and its downstream caspase 3, we found that both caspases were cleaved after curcumin treatment, but cleavage started earlier and was higher in NT1-M than in NT1-WT CLL cells (N = 4; **Figure 2D**).

Also, in NT1-M, but not in NT1-WT CLL cells, curcumin-induced apoptosis was preceded by a rapid and significant increase of [Ca^2+^]_i_ levels compared to DMSO (N = 6, *P* = 0.031) (**Figure 2E**). In keeping with this observation, it has been shown that [Ca^2+^]_i_ accumulation induced by high curcumin doses is accompanied by increased expression of UPR markers, including CHOP (35). Altogether, these results suggest that CLL harboring *NOTCH1* mutation tends to be more sensitive to induced ER stress.

### *NOTCH1* mutation was associated with an increased ER stress-induced apoptosis rate after curcumin treatment

**Figure 3:**
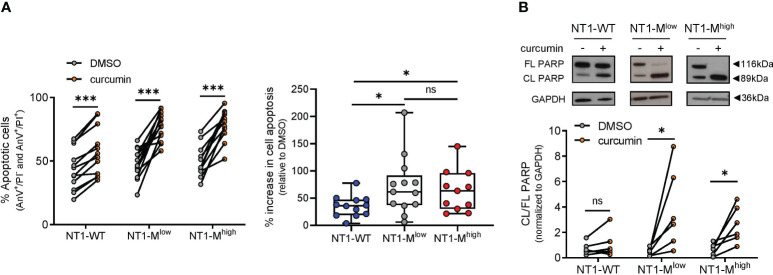
NT1-M CLL cells are more susceptible than NT1-WT to curcumin-induced apoptosis. Primary CLL NT1-WT (N = 12); NT1-M with a mutation allelic burden ranging from 0.03% to 12%, referred to as NT1-M^low^ (N = 13); and NT1-M with a mutation allelic burden higher than 12%, referred to as NT1-M^high^ (N = 11), were incubated with curcumin (15 µM) or DMSO (0.05%) for 24 h. **(A)** Apoptosis was assessed by annexin V/PI (AnV/PI) assay, which allows distinguishing viable (AnV^−^/PI^−^), early apoptotic (AnV^+^/PI^−^), late apoptotic (AnV^+^/PI^+^), and necrotic cells (AnV^−^/PI^+^). Dot-and-line diagram (left) of apoptotic (early plus late) cells. ***P< 0.001, according to Wilcoxon paired test. Box and whisker with data points (right) of the percentage increase in the apoptosis of curcumin-treated NT1-WT, NT1-M^low^, and NT1-M^high^ CLL cells, normalized to their respective DMSO controls. Data are presented as mean ± SD. *P< 0.05; ns, not significant, according to Mann–Whitney unpaired test. **(B)** Representative Western blot (top) showing full length (FL) and cleaved (CL) PARP and dot-and-line diagram of CL/FL PARP ratio (bottom) in NT1-WT, NT1-M^low^, and NT1-M^high^ CLL cells (N = 6, per group), incubated with curcumin or DMSO. *P< 0.05; ns, not significant, according to Wilcoxon paired test.

We then analyzed the effect of curcumin treatment for 24 h on the apoptosis of NT1-WT (N = 12), NT1-M^low^ (N = 13), and NT1-M^high^ (N = 11) CLL cells, using annexin V/PI assay. As shown in **Figure 3A**, curcumin treatment increased the apoptosis of NT1-WT (*P* = 0.0005), NT1-M^low^ (*P* = 0.0002), and NT1-M^high^ (*P* = 0.001) cells compared with that of the DMSO control. However, the percentage of increase in cell apoptosis induced by curcumin and normalized to that of the respective DMSO control was higher in both NT1-M^low^ (71.8% ± 53.1% vs. 35.1% ± 19.3%; *P* = 0.034) and NT1-M^high^ (63.3% ± 38.4% vs. 35.1% ± 19.3%; *P* = 0.05) than in NT1-WT CLL (**Figure 3A**, right). These results suggest that CLL cells harboring *NOTCH1* mutations appeared to be more prone to apoptosis than NT1-WT cells, regardless of mutational burden.

In line with flow cytometry data, WB analysis of the apoptotic marker PARP showed that its cleavage was increased by curcumin in NT1-M^low^ and NT1-M^high^ cells (*P* = 0.031, both) compared to that in the DMSO control (N = 6, for each group), whereas in NT1-WT cells (N = 6), curcumin did not significantly change PARP cleavage compared to that in DMSO (*P* = 0.218; **Figure 3B**).

### NOTCH1 signaling and MCL1 protein expression were downregulated by curcumin in *NOTCH1*-mutated CLL

**Figure 4:**
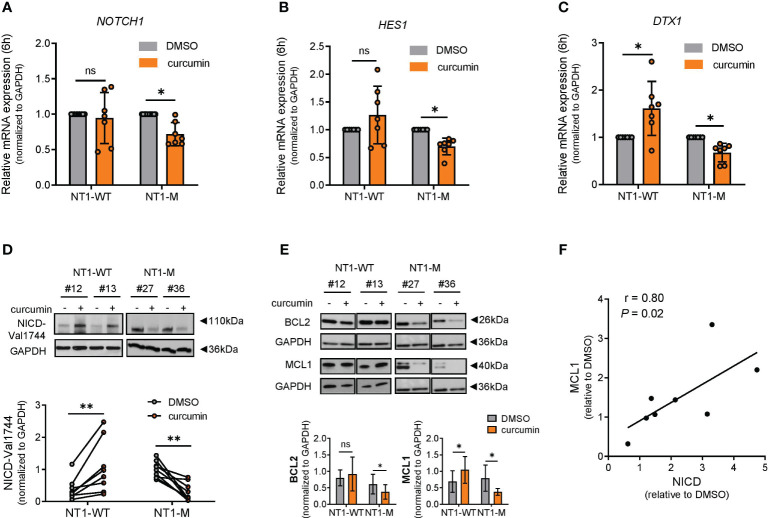
NOTCH1 is downregulated by curcumin in NT1-M cells and correlates with MCL1 expression. **(A)** Bar graphs with data points show the gene expression of *NOTCH1* mRNA expression in NT1-WT and NT1-M (N = 7 for both) assessed by qPCR, after 6 h of 15 μM curcumin or DMSO (0.05%) treatment. **(B, C)** Bar graphs with data points show the gene expression of NOTCH1 downstream targets *HES1* and *DTX1*, after 6 h of curcumin or DMSO treatment (N = 7). mRNA levels were normalized to GAPDH and are represented as fold change by using the DMSO control as a reference set to 1. **(D)** Representative Western blot (top) and dot-and-line diagram (bottom) analysis of NICD-Val1744 in NT1-WT and NT1-M CLL cells (N = 8 and N = 9, respectively) incubated with DMSO (0.05%) or 15 μM curcumin for 24 h. **(E)** Representative Western blot (top) and bar graph (bottom) analysis of MCL1 (N = 7, both groups) and BCL2 protein levels (N = 6, both groups), after 24 h of curcumin or DMSO exposure. **(F)** Correlation analysis between NICD-Val1744 and MCL1 expression in NT1-WT cells after treatment. Data are presented as mean ± SD. **(A–E)** *P< 0.05, **P< 0.01; ns, not significant as determined by Wilcoxon paired test. **(F)** The correlation between MCL1 and NICD values was assessed by Spearman’s correlation test (r).

To investigate whether ER stress induced by curcumin in NT1-M CLL cells was associated with modulation of NOTCH1 signaling, we first measured *NOTCH1* mRNA expression in primary CLL cells after 6 h of curcumin exposure. The *NOTCH1* gene expression was significantly reduced in NT1-M cells (N = 7; *P* = 0.015) compared to that in the DMSO control but not significantly changed in NT1-WT cells (N = 7; *P* = 0.68) (**Figure 4A**). Accordingly, **Figures 4B, C** show that after curcumin treatment, there was a significant reduction in the mRNA expression of NOTCH1 downstream target genes *HES1* and *DTX1* in NT1-M (N = 7; *P* = 0.015, for both) compared to that in the DMSO control, respectively. Conversely, in NT1-WT CLL, curcumin induced a significant increase in *DTX1* mRNA compared to that in DMSO (*P* = 0.031) but did not significantly affect *HES1* levels. Next, we evaluated the effect of curcumin treatment for 24 h on the levels of the active NICD protein. As shown in **Figure 4D**, in NT1-M cells, NICD levels were significantly decreased by curcumin compared to that in the DMSO control (N = 9; *P* = 0.004) and were negatively correlated with the *NOTCH1* mutation allelic ratio (*P* > 0.0001; **Supplementary Figure S4**). Conversely, in NT1-WT, curcumin induced a significant increase in NICD levels compared to that in the DMSO control (*P* = 0.0078). It has been shown that both NOTCH1 signaling and prolonged ER stress are associated with the expression of anti-apoptotic BCL2 family members, such as MCL1 (11, 36). WB analysis showed that in NT1-M CLL, curcumin reduced the levels of both MCL1 (*P* = 0.015) and BCL2 (*P* = 0.031) compared with those in DMSO, whereas in NT1-WT cells, it increased the levels of MCL1 (*P* = 0.015) without significantly influencing BCL2 expression (**Figure 4E**). In NT1-WT, the increase in MCL1 expression correlated positively with the increase in NICD levels (*P* = 0.021; **Figure 4F**), reinforcing the relationship between NOTCH1 signaling and sustained MCL1 levels in CLL cells (37).

### The apoptotic effect of the BCL2 inhibition on *NOTCH1*-mutated CLL was potentiated by curcumin

**Figure 5:**
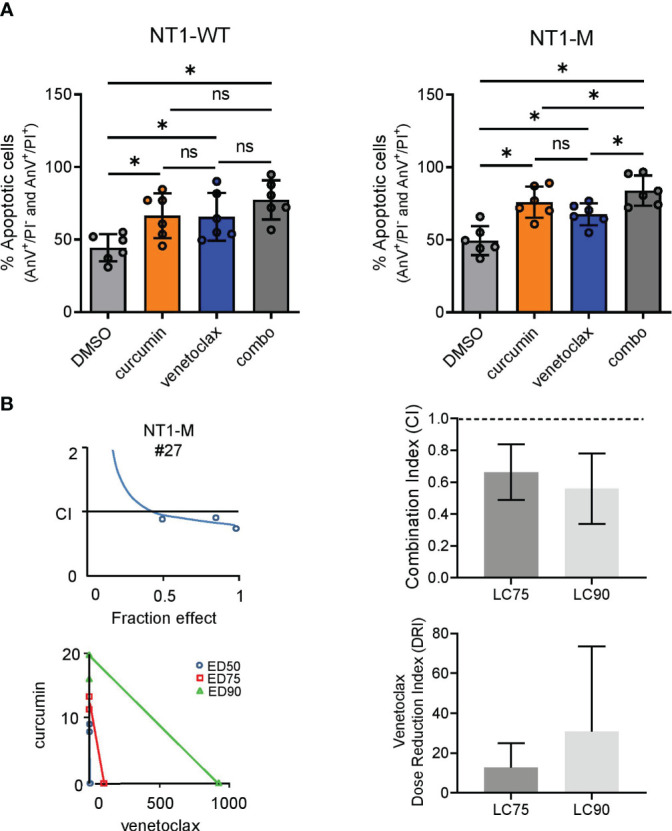
Venetoclax synergizes with curcumin in NT1-M CLL cells. **(A)** AnV/PI assay was used to assess the percentage of apoptotic (early plus late) NT1-WT and NT1-M (N = 6, for both) CLL cells treated for 24 h with curcumin (15 μM), venetoclax (2 nM), curcumin/venetoclax combination, or DMSO (0.05%) as control. Data are presented as mean ± SD. *P< 0.05; ns, not significant as determined by Wilcoxon paired test. **(B)** CI curves and isobolograms computed by the Chou–Talalay model (CalcuSyn software, Biosoft, Cambridge) from the dose–effect profiles of activated leukemic cells treated for 24 h with increasing concentrations of curcumin (7.5–30 μM), venetoclax (1–4 nM), or venetoclax/curcumin at a constant ratio (1:7.5). CI measures drug interaction effects: additive: 0.9 ≤ CI ≤ 1.1; synergism: CI< 0.9; and antagonism: CI > 1.1. Isobolograms: the x- and y-axes represent the doses of venetoclax and curcumin, respectively. The intercepts of the three lines on the x- and y-axes represent the dose of the same efficacy when the two drugs are used alone, which are here expressed as half, 75%, and 90% effective doses (i.e., ED50, ED75, and ED90, respectively). Additive: point on the line; synergism: point below the line; antagonism: point above the line. CI values at the “fractional effect levels” LC75 and LC90 (concentrations lethal to 75% and 90% of CLL cells, respectively). The dotted lines indicate CI = 0.9 and CI = 1.1. Data are presented as mean ± standard deviation (SD). *P< 0.05; ns, not significant as determined by using one-way ANOVA (Geisser–Greenhouse correction).

MCL1 expression and NOTCH activation also contribute to resistance to venetoclax therapy in CLL (38, 39). We tested the combined effect of curcumin (15 μM) and venetoclax (2 nM) on apoptosis, evaluated by AnV/PI assay, of NT1-M and NT1-WT CLL cells (N = 6, for both) after 24-h treatment. As shown in **Figure 5A**, in NT1-WT cells, the apoptotic effect induced by curcumin or venetoclax as single agents, compared to that in the DMSO control, was not significantly increased when the agents were used in combination. Conversely, in NT1-M cells, curcumin combined with venetoclax significantly increased the apoptotic effect induced by each single agent (*P* = 0.031 for both; **Figure 5A**). The CI and isobologram of three NT1-M patients demonstrated a synergic effect for curcumin/venetoclax combination (**Figure 5B**, **Supplementary Figure S5**). Based on the dose–effect curves of drugs alone and in combination, we calculated the DRI; we found that curcumin sensitized NT1-M CLL cells to the cytotoxic action of venetoclax and significantly decreased the efficacious dose of venetoclax.Curcumin elicited anti-leukemic effects associated with NOTCH1 inhibition in Eμ-TCL1 mice.

**Figure 6:**
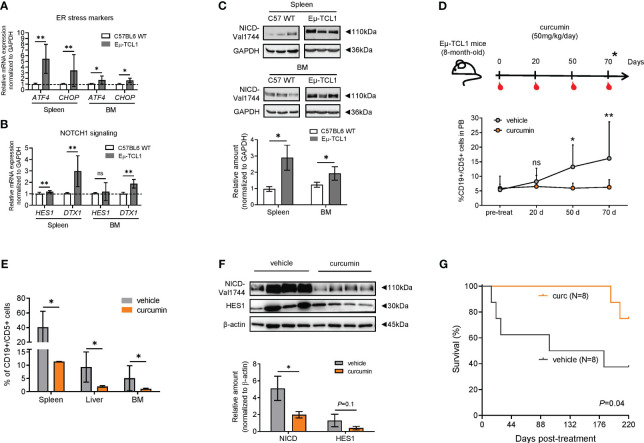
Curcumin suppresses the development of leukemic cells (CD19^+^/CD5^+^) and reduces NOTCH1 signaling in Eμ-TCL1 mice. **(A)** qPCR analysis of *ATF4* and *CHOP* in B cells from the spleen or BM from C57BL/6 WT (N = 6) or Eμ-TCL1 mice (N = 6). **(B)** qPCR analysis of *HES1* and *DTX1* mRNA levels in B cells from the spleen or BM of C57BL/6 or Eμ-TCL1 mice. **(C)** Western blot analysis and bar graph of NICD. **(D)** Eight-month-old Eμ-TCL1 mice were injected intraperitoneally with curcumin (50 mg/kg/day) dissolved in a vehicle (corn oil) or with vehicle alone daily for 2 months and evaluated according to the scheme depicted (upper panel). The expansion of CD19^+^/CD5^+^ cells in the PB (N = 4 per group) was assessed by flow cytometry at the indicated time points (bottom panel). **(E)** Accumulation of CD19^+^/CD5^+^ cells in the spleen, liver, and BM of Eμ-TCL1 mice treated with curcumin or vehicle (N = 4, per group) assessed by flow cytometry. **(F)** Western blot analysis of CD19^+^/CD5^+^-sorted cells of BM, indicating the significant reduction of NICD expression (N = 4, per group) and a tendency of reduction for HES1 (N = 3, per group). Data are presented as mean ± SD. **(A-F)** *P< 0.05, **P< 0.01; ns, not significant as determined by unpaired t-test. **(G)** Kaplan–Meier survival plot of Eμ-TCL1 mice treated with curcumin (N = 8) or vehicle alone (N = 8) from two independent experiments. Overall survival was determined by using Kaplan–Meier curves and log-rank (Mantel–Cox) test.

To better define the association between NOTCH1 signaling and ER stress, we used Eμ-TCL1 mice, a widely used mouse model for CLL, displaying an aberrant response to ER stress (14). B cells from the spleen and BM of homozygous Eμ-TCL1 mice expressed increased mRNA levels of *ATF4* (*P* = 0.002, spleen; *P* = 0.04, BM) and *CHOP* (*P* = 0.002, spleen; *P* = 0.047, BM) (**Figure 6A**), as well as higher NOTCH1 downstream targets *HES1* (N = 6; *P*< 0.01, spleen) and *DTX1* (N = 6; *P*< 0.01, spleen; *P*< 0.01, BM) (**Figure 6B**) and NICD protein expression (P = 0.01, spleen; P = 0.05, BM; **Figure 6C**), compared to B cells from C57BL6 WT and heterozygous Eμ-TCL1 mice (**Supplementary Figure S6**).To examine whether curcumin was able to slow down the progression of CLL in Eμ-TCL1 mice, we treated mice with curcumin (50 mg/kg once every 2 days for 70 days) or vehicle (**Figure 6D**). While CD19^+^/CD5^+^ leukemic cells progressively expanded in the PB of vehicle-treated mice, curcumin treatment prevented their expansion, and this might be due to their higher apoptosis levels (**Supplementary Figure S7**).

**Figure 6E** shows that CD19^+^/CD5^+^-infiltrating cells were significantly reduced in the spleen, liver, and BM (*P* = 0.05, *P* = 0.028, *P* = 0.028, respectively) of curcumin-treated mice. Splenic dimensions did not show a reduction when compared to those of the control group (**Supplementary Figure S8**). Analysis of NOTCH1 activation in BM CD19^+^/CD5^+^ cells showed that NICD levels were reduced in curcumin-treated mice compared to those in vehicle-treated mice (N = 4, *P* = 0.028, **Figure 6F**). The Kaplan–Meier survival curve revealed that vehicle-treated mice (N = 8, median survival = 142 days) succumbed significantly earlier than curcumin-treated mice (N = 8, m. s. = undefined, *P =* 0.04, **Figure 6G**).

## Discussion

A hallmark of several human cancers is UPR activation, a multifunctional pathway that can either enable cells to survive by adapting to adverse environmental conditions (40) or evolve into a cell death program in case of severe/prolonged stress or when the regulatory mechanisms of UPR are altered (41, 42). The ER stress response has been found to be essential for the survival of CLL cells (6), but the core mechanisms are not fully understood. Here, we investigated the relationship between dysregulated NOTCH1 signaling and the ER stress/UPR pathway in CLL.

Our bioinformatics analysis revealed the enrichment of UPR genes in NT1-M CLL cells, mostly involving the PERK branch, such as *ATF4* and *CHOP*. In keeping with these data, we demonstrated that PGA1 cells overexpressing mutation of *NOTCH1* showed an increase in *ATF4* and *CHOP* gene expression when compared with PGA1 cells transduced with the empty vector and with NT1-WT. These results suggest that *NOTCH1* mutation is associated with sustaining *ATF4* and *CHOP* gene expression, although the underlying mechanisms for that response remain unknown.

Overexpression of ATF4 has been linked to cancer cell proliferation, invasion (43), stemness, poor prognosis (44), higher tumor grade, therapy resistance, and shorter survival (45) in a variety of cancers. However, under severe ER stress, ATF4 can also promote cell death by activating CHOP transcription, which induces multiple pro-apoptotic genes and suppresses the synthesis of the anti-apoptotic BCL2 proteins (10, 11).

Based on the above observations, in this work, we tested the response of primary CLL cells with dysregulated NOTCH1 to ER stress induction by using curcumin, a natural agent known to promote apoptosis of primary CLL cells and to lead to ER stress in other types of cancer (33–36). We found that NT1-M CLL cells showed, compared with NT1-WT, a heightened ER stress-mediated apoptosis characterized by high expression of ATF4, CHOP, BiP/HSPA5, and caspase 4 cleavage.

Strikingly, in CLL with *NOTCH1* mutation, the induction of this apoptotic pathway by curcumin was preceded by a rapid increase of [Ca^2+^]_i_ levels, indicating that the release of Ca^2+^ from the ER may activate the apoptotic effectors. Sala de Oyanguren et al. (2020) showed that an increase in curcumin doses causes accumulation of [Ca^2+^]_i_ and a concomitant increase in the expression of UPR markers, such as CHOP (46). Additionally, recent studies using the HL-60 and BCPAP tumorigenic cell lines showed that a major fraction of curcumin uptake localizes in the ER, causing prolonged UPR preceded by a calcium status change (33, 35).

The calcium disruption effect of curcumin is mainly attributed to the inhibition of the sarco/ER calcium ATPase (SERCA), which is responsible for transporting ions from the cytosol back to the ER (47, 48). In this context, Roti et al. (2018) and Marchesini et al. (2020) demonstrated that the SERCA selective modulators JQ-FT and CAD204520, respectively, preferentially target cells harboring gain-of-function *NOTCH1* mutations over WT in T-ALL (49, 50).

**Figure 7:**
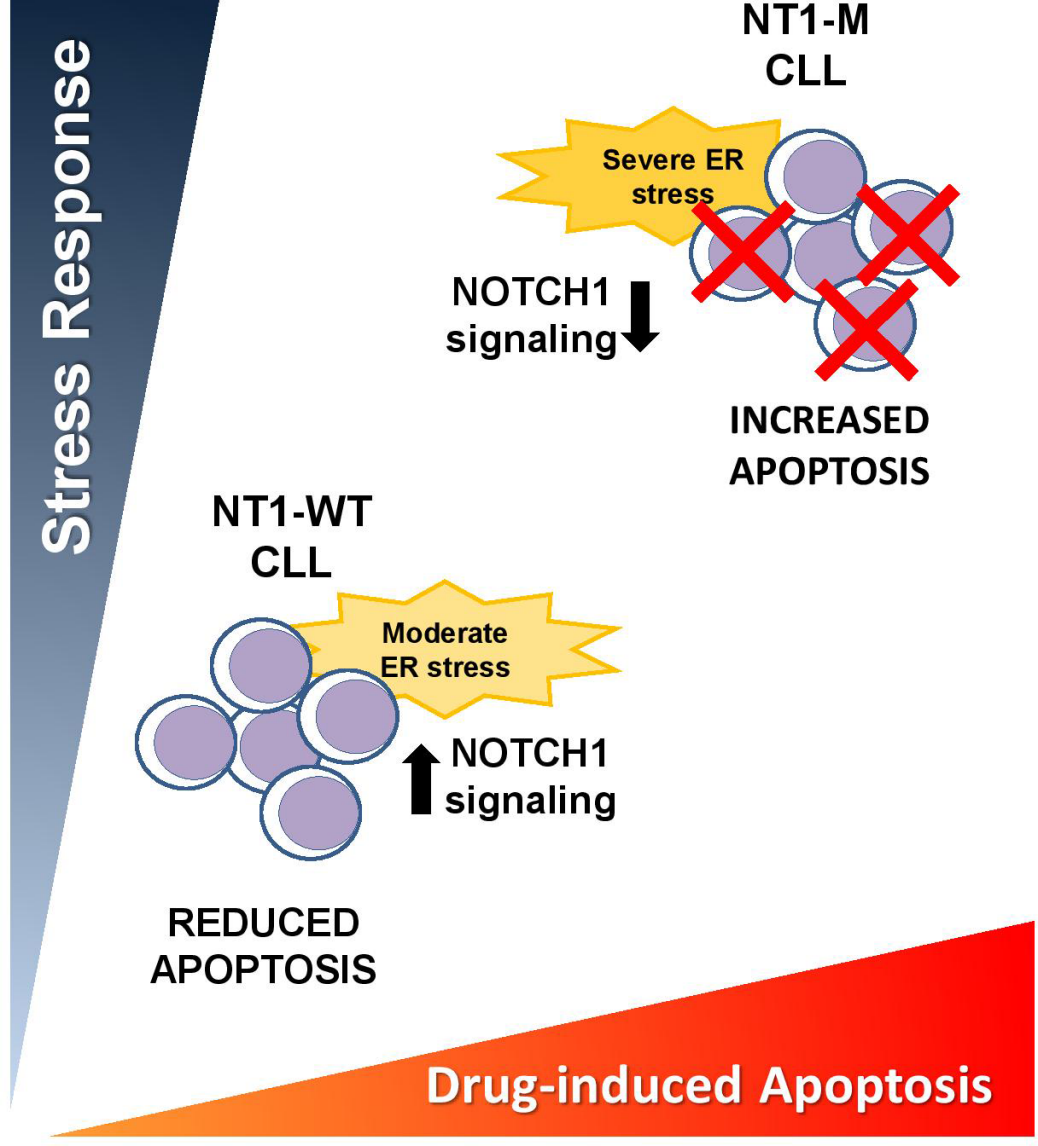
Schematic diagram summarizing our working model. Hypothetical association of ER stress/NOTCH1 signaling with susceptibility to ER stress-induced apoptosis in CLL with dysregulated NOTCH1. CLL cells harboring *NOTCH1* mutation (NT1-M) show higher basal levels of ER stress than *NOTCH1*-WT (NT1-WT) cells. ER stress-inducing drugs further increase ER stress in NT1-M CLL cells and induce high levels of apoptosis, which are accompanied by decreased NOTCH1 signaling activation. Conversely, NT1-WT CLL cells respond to ER stressors with a moderate ER stress, low levels of apoptosis, and increased NOTCH1 activation, which probably serves to counteract ER stress.

The high apoptotic levels induced by curcumin in NT1-M CLL cells were also associated with a drastic reduction of NOTCH1 signaling, as shown by the decreased levels of the active NICD and its downstream targets *HES1* and *DTX1*. In line with these findings, experiments with a derivative of the SERCA inhibitor thapsigargin demonstrated that T-ALL cells harboring *NOTCH1* mutation showed a marked reduction in the NOTCH1 signaling pathway (50). Additionally, previous research has shown that high ER stress induced by γ-secretase inhibitors in CLL causes apoptosis, which is followed by NOTCH1 signaling suppression in CLL (13). Conversely, in this study, we found that the low levels of ER stress-related apoptosis induced by curcumin in NT1-WT CLL cells were accompanied by enhanced NOTCH1 signaling activation. The results indicate that increased NICD might serve to counteract ER stress, thus favoring a prosurvival UPR, as schematically shown in **Figure 7**.

ER stress-induced apoptosis also depends on the core mitochondrial apoptosis signaling regulated by the BCL2 protein family (51). Specifically, we found that NT1-M cells showed a drastic reduction in MCL1 protein expression after treatment with curcumin. In contrast, NT1-WT cells showed an increase in MCL1 levels, which might be related to controlled ER stress and reduced apoptosis. These results, along with our previous evidence that NOTCH1 signaling sustains MCL1 expression to promote CLL cell survival (37), further suggest that the NOTCH1/MCL1 axis could be the barrier to apoptosis induced by ER stressors.

It has been shown that high levels of MCL1 expression in CLL cells also represent an important mechanism of resistance to venetoclax therapy (39). In this context, we demonstrated that the combination of curcumin and venetoclax showed improved anti-leukemic effects compared to a single curcumin treatment in NT1-M CLL cells. These results suggest that the downregulation of the NOTCH1/MCL1 axis induced by a prolonged ER stress sensitized NT1-M CLL cells to the cytotoxic action of venetoclax, by improving its therapeutic effect. This is also demonstrated by the evidence that the DRI of venetoclax appears to be higher when combined with curcumin.

In order to get further insight into the role of NOTCH1 signaling in ER stress conditions *in vivo*, we used Eμ-TCL1 mice, since chronic overexpression of TCL1 has been demonstrated to activate the ER stress response for malignant progression of CLL (14, 52). Strikingly, we observed that B cells from Eμ-TCL1 mice showed increased levels of NICD and its targets *HES1* and *DTX1* when compared to those from control mice. This finding indicates that TCL1 may contribute to ER stress and CLL progression even with enhanced NOTCH1 signaling and reinforces the coexistence of an ER-stressed environment and increased NOTCH1 activation (53).

Furthermore, we found that CD19^+^/CD5^+^ cells from Eμ-TCL1 mice, after *ex vivo* curcumin exposure, showed increased ATF4 and CHOP expression levels, suggesting that curcumin potentiates ER stress even in murine leukemic cells. Additionally, after 2 months of treatment with curcumin, Eμ-TCL1 mice showed a reduced percentage of CD19^+^/CD5^+^ cells in PB and infiltrated organs, accompanied by prolonged overall survival compared to untreated mice. This anti-leukemic effect of curcumin correlated with reduced NICD levels in CD19^+^/CD5^+^ cells from the BM of treated mice compared with those of untreated mice. Given the key role of NOTCH1 in CLL onset and progression (54), it is likely that in curcumin-treated Eμ-TCL1 mice, impaired NOTCH1 signaling, associated with ER stress exacerbation, could be implicated in slowing down CLL development and in improving the survival of leukemic mice. The absence of an *in vivo* model of *NOTCH1* mutation represents a significant limitation in our study, restricting a more comprehensive exploration of the complex interaction between increased NOTCH1 signaling and treatment with ER stressors such as curcumin. Nevertheless, further ER stress stimulation in NOTCH1-deregulated CLL might represent an additional therapeutic opportunity for these high-risk patients and improve the therapeutic effect of drugs currently used in CLL.

## Conclusion

Overall, this study highlights that *NOTCH1* mutation is associated with the expression of specific ER stress-associated markers in CLL and renders CLL cells more susceptible to ER stress-mediated apoptosis. These altered ER stress conditions, likely due to the high proliferation and protein synthesis rates induced by NOTCH1 signaling stabilized by mutation, might be unable to engage a protective UPR able to counteract a prolonged ER stress, as instead occurs in NT1-WT cells. Importantly, our results indicate that ER stress induction in NT1-M CLL might represent an additional therapeutic opportunity for these high-risk patients and improve the therapeutic effect of drugs currently used in CLL.

## Data availability statement

The original contributions presented in the study are included in the article/**Supplementary Material**. Further inquiries can be directed to the corresponding author.

## Ethics statement

The studies involving humans were approved by University of Perugia, Perugia, Italy (approval 2015–001). The studies were conducted in accordance with the local legislation and institutional requirements. The participants provided their written informed consent to participate in this study. The animal study was approved by Italian Health Ministry (authorization no. 1155/2015-PR and no. 971/2020-PR). The study was conducted in accordance with the local legislation and institutional requirements.

## Author contributions

ECSB and PS conceived and designed the project. CR and FMA performed *in vivo* study and analysis. ECSB and VT designed and performed *in silico* analysis. CR, FMA, ED, FF, SC, BP, SB, MN, AE, AS, and RA contributed to molecular experiments, Western blots, and interpretation of data. DS acquired and analyzed flow cytometric data. ECSB, PS, ER, and FI prepared, wrote, reviewed, and/or revised the manuscript. All authors contributed to the article and approved the submitted version.
