# Supplementary material for: NOTCH1-mutated chronic lymphocytic leukemia displays high endoplasmic reticulum stress response with druggable potential
Source: Front Oncol. 2023 Sep 25;13:1218989. doi: 10.3389/fonc.2023.1218989 (PMC10561002; doi:10.3389/fonc.2023.1218989)
Supplement: Supplementary file 1 [file DataSheet_1.pdf]

Supplementary Information

NOTCH1-mutated CLL displays high endoplasmic reticulum stress response with druggable potential

Silva Barcelos *et al*.

Contents:

- Supplementary Tables:

Supplementary Table S1: Characteristics of studies included in the bioinformatics analysis

Supplementary Table S2: List of antibodies

Supplementary Table S3.xls: Sequences of primers used for RT-PCR

Supplementary Table S4.xls: Genes differentially expressed among studies (pfp<0.05)

Supplementary Table S5.xls: Gene ontology analysis with deregulated genes of *NOTCH1*-M versus -WT

Supplementary Table S6.xls: Reactome Pathways analysis with deregulated genes of *NOTCH1*-M versus –WT

Supplementary Table S7.xls: CLL patients characteristics

- Supplementary Figures:

Supplementary Figure S1. Pipeline steps for bioinformatics study.

Supplementary Figure S2. PGA1 cells transduced with FG12-based lentiviral vector expressing GFP, containing plasmid pNICD WT or pNICD mutated (c.7541_7542delCT), or empty, used as mock control.

Supplementary Figure S3. Gene expression of the ER/UPR markers of primary CLL cells after induction of thapsigargin, for 6h.

Supplementary Figure S4. Linear regression graph plot showed correlation between the expression of NICD of curcumin-treated/DMSO-treated primary CLL cells and Allelic Ratio assessed by ddPCR.

Supplementary Figure S5. Combination index (CI) curves and isobolograms of NT1-M primary CLL cells treated with curcumin.

Supplementary Figure S6. Levels of western blot of NICD in Eμ-TCL1 mice.

Supplementary Figure S7. Percentage of AnnexinV+/CD19+/CD5+ cells from Peripheral Blood (PB) of Eμ-TCL1 mice treated with vehicle or curcumin, after 70 days.

Supplementary Figure S8. Spleen dimensions of vehicle and curcumin-treated Eμ-TCL1 mice.

Supplementary Table S1. Characteristics of studies included in the bioinformatics analysis

GEO accession no.	Total of Samples	*NOTCH1* WT/M	Sample source	Platform	PMID
GSE92626	N = 57	12/10 (6*; 2 (3'-UTR); p.Q2417X and p.Q2488X)	Whole Blood	Illumina HiSeq 2500	28314854 (Fabbri G, Holmes AB, Viganotti M, Scuoppo C et al., 2017)
GSE75122	N = 10	5/5*	Whole blood	Agilent-014850 Whole Human Genome Microarray 4x44K G4112F	28321119 (Pozzo F, Bittolo T, Vendramini E, Bomben R et al., 2017)
GSE137024	N = 28	13/6*	Whole blood	Agilent-039494 SurePrint G3 Human GE v2 8x60K Microarray 039381	33121237 (Pozzo F, Bittolo T, Tissino E, Vit F et al., 2021)

GEO: Gene Expression Omnibus. *(c.7541_7542delCT)

Supplementary Table S2. List of antibodies

Target protein	Clone	Target Species	Supplier	Catalog number	Application
CD45	J33	Human	Beckman Coulter	B36294	Flow cytometry
CD5	BL1a	Human	Beckman Coulter	A21690	Flow cytometry
CD19	J3-119	Human	Beckman Coulter	A07770	Flow cytometry
CD11b	Bear1	Human	Beckman Coulter	B36295	Flow cytometry
CD3	UCHT1	Human	Beckman Coulter	B49204	Flow cytometry
CD5	53-7.3	Mouse	Miltenyi Biotec	130-101-960	Cell sorting
CD19	6D5	Mouse	Miltenyi Biotec	130-119-800	Cell sorting
BiP/HSPA5	40	Human/Mouse	BD Transduction Laboratories	610979	WB
CHOP/GADD153	B-3	Human/Mouse	SCB	sc7351	WB
Caspase-3	Polyclonal	Human	CST	#9662	WB
Caspase-4	4B9	Human	MBL	M029-3	WB
PARP	Polyclonal	Human	CST	#9542	WB
Cleaved NOTCH1 (Val1744, ICN)	D3B8	Human/Mouse	CST	#4147	WB
MCL1	D35A5	Human/Mouse	CST	#5453	WB
BCL2	124	Human	Dako	M0887	WB
ATF4	D4B8	Human/Mouse	CST	#11815	WB
HES1	E-5	Human/Mouse	SCB	sc-166410	WB
IRE1α	14C10	Human/Mouse	CST	#3294	WB
eIF2α	-	Human/Mouse	CST	#9722	WB
ATF6	D4Z8V	Human/Mouse	CST	#65880	WB
Laminin	B-4	Human/Mouse	SCB	sc-55605	WB
GAPDH	GAPDH-71.1	Human/Mouse	Sigma	G8795	WB
β-actin	AC-15	Human/Mouse	Novus	NB 600-501	WB

Abbreviations: CST, Cell Signaling Technology; SCB, Santa Cruz BioTechnologies WB, western blot;

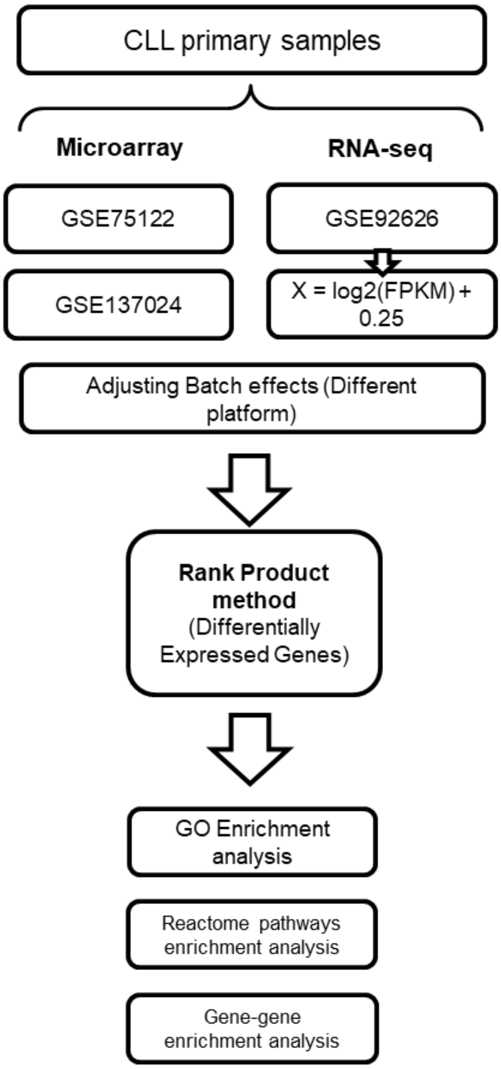

Supplementary Figure S1. Pipeline steps for bioinformatics study. The gene expression information of both *NOTCH1*-WT (NT1-WT) and *NOTCH1*-mutated (NT1-M) CLL samples was acquired from the Gene Expression Omnibus (GEO) database. To ensure data quality and comparability, we conducted data processing, including removal of duplicate samples, normalization, and batch effect correction. The Batch effect was adjusted using SVA package to remove any systematic variations introduced by different studies. To identify DEGs from the comparison between NT1-WT and NT1-M CLL samples, we employed the Rank Product method. This non-parametric statistical approach is robust to handle heterogeneous datasets and effectively captures subtle but consistent changes in gene expression across different studies. We computed the Rank Product values for each gene, and the significance of the differential expression was

determined based on permutation tests. Gene Ontology (GO) enrichment analyses and pathway analysis were performed using g:Profiler and Reactome database, respectively. Gene regulatory networks was constructed using the GeneMania tool from Cytoscape software.

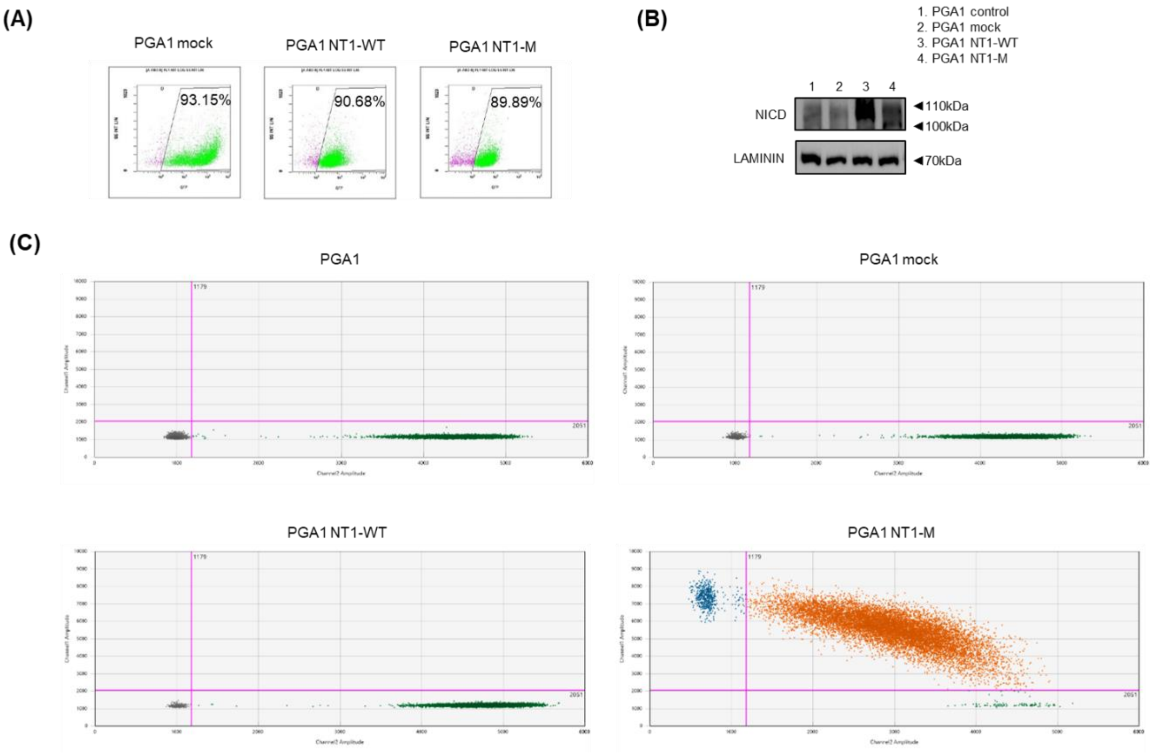

Supplementary Figure S2. PGA1 cells transduced with FG12-based lentiviral vector expressing GFP, containing plasmid pNICD WT or pNICD mutated (c.7541_7542delCT), or empty, used as mock control. (A) Plots depicting the proportion of GFP-positive cells among PGA1-sorted cells in the respective transduced cell populations. (B) Western Blot analysis of NICD in untransduced PGA1 cells (control) and PGA1-transduced cells (PGA1 mock, NT1-WT, and NT1-M). Laminin was used as a protein loading control. (C) Absolute quantification of ddPCR multiplex assay from unstransduced PGA1 cells and transduced PGA1 cells. The two-dimensional cluster plot in which Channel 1 fluorescence (FAM-labelled probe for NICD mutated) was plotted against Channel 2 (HEX-labelled probe for NICD WT). Clusters of droplets arranged in an orthogonal arrangement to depict different types: FAM/HEX negative droplets (double-negative,

grey), FAM positive droplets (blue), HEX positive droplets (green), and FAM/HEX positive droplets (double-positive, orange).

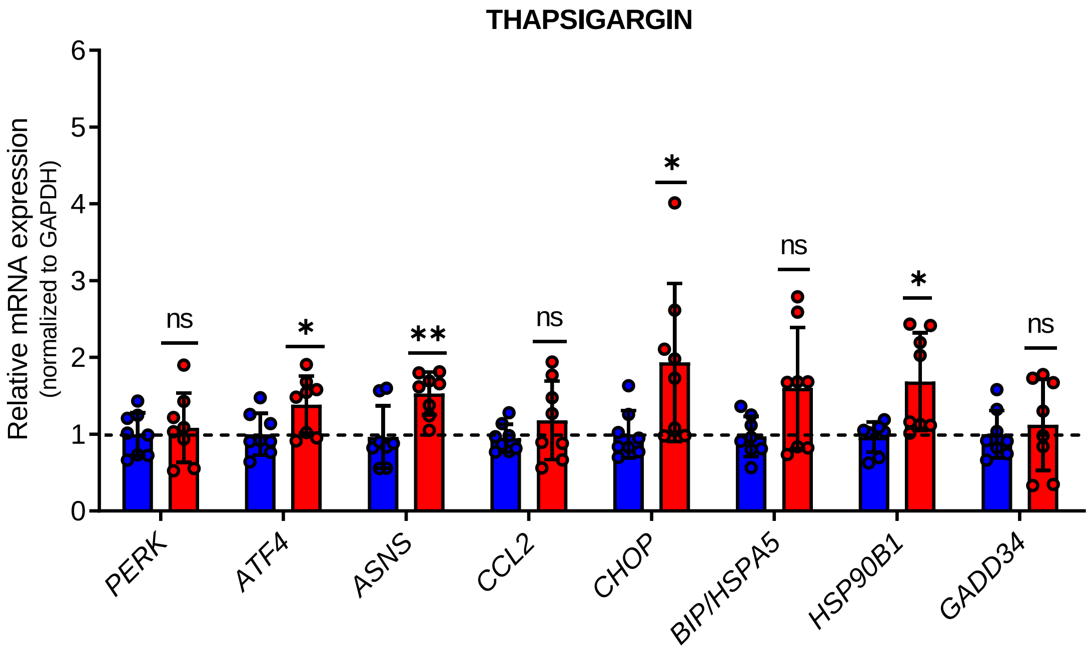

Supplementary Figure S3. Gene expression of the ER/UPR markers of primary CLL cells after induction of thapsigargin, for 6h. Bar graphs of ER/UPR markers gene expression from NT1-WT (N=8, blue points) and NT1-M (N=8, red points) cells after 6h of treatment with thapsigargin (1µM), a chemical inducer of ER stress. mRNA levels were normalized to GAPDH and represented as fold change by DMSO-treated as a reference set to 1 (dashed line). *P<0.05, **P<0.01, ns, not significant as determined by Wilcoxon paired test.

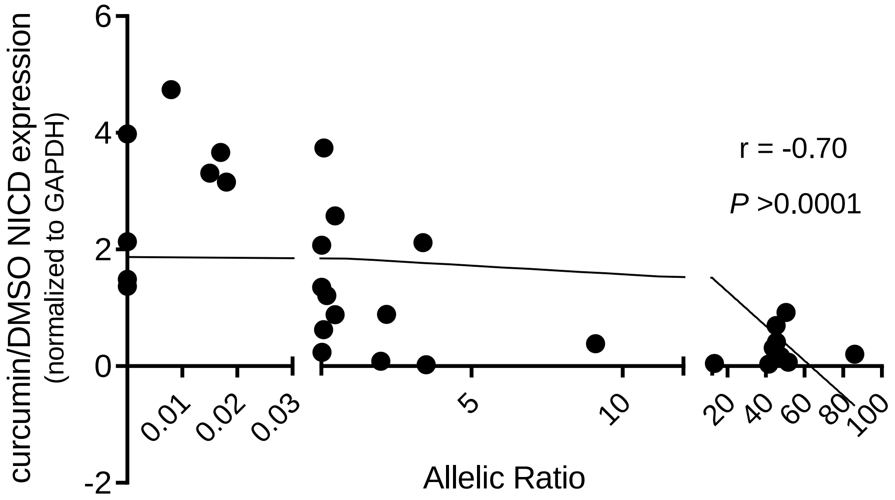

Supplementary Figure S4. Linear regression graph plot showed correlation between the expression of NICD of curcumin-treated/DMSO-treated primary CLL cells and Allelic Ratio assessed by ddPCR. The graph shows points for the results of NT1-WT (N=8), NT1-M^{low} (N=13) and NT1-M^{high} (N=9) CLL cells. The correlation between NICD Ratio values and % of Allelic Ratio was calculated by Spearman's correlation test (r).

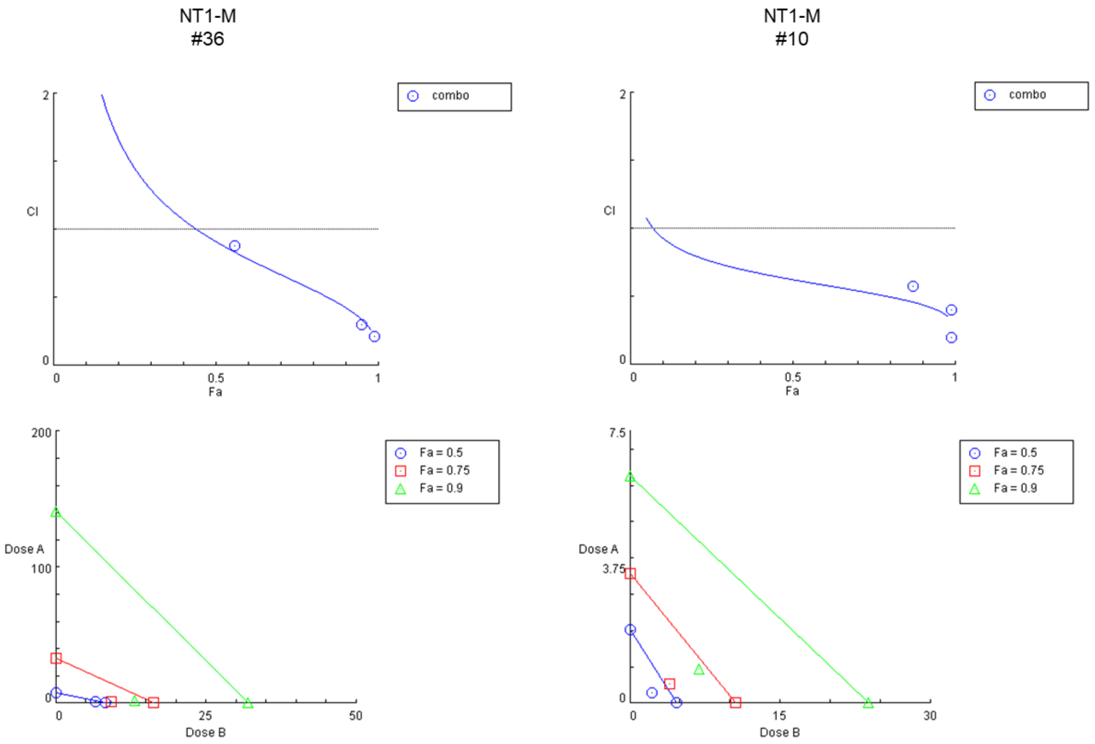

	LC75	LC90
CI	0.611	0.424
DRI venx	26.765	80.085

	LC75	LC90
CI	0.520	0.438
DRI venx	6.791	6.767

Supplementary Figure S5. Combination index (CI) curves and isobolograms of NT1-M primary CLL cells treated with curcumin. CI and Isobolograms computed by the Chou–Talalay model (CalcuSyn software, Biosoft, Cambridge) from dose–effect profiles of primary CLL cells treated for 24 h with increasing concentrations of venetoclax (1–4 nM), curcumin (7.5–30 μM) or venetoclax/curcumin at constant ratios (1:7.5). CI values at the 'fractional effect levels' LC75 and LC90 (concentration lethal to 75% and 90% of CLL cells, respectively). CI and Dose Reduction Index (DRI) values at LC75 and LC90 of CLL patients.

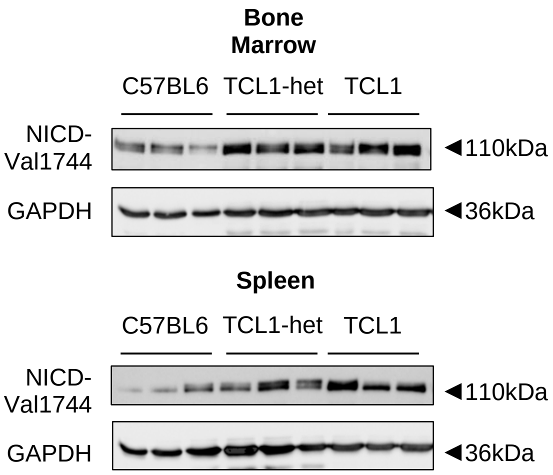

Supplementary Figure S6. Levels of western blot of NICD in Eμ-TCL1 mice. Densitometric data of levels of NICD in sorted CD19+ cells from bone marrow and spleen from C57BL6 WT, TCL1 heterozygous and TCL1 homozygous mice. GAPDH was used as a protein loading control.

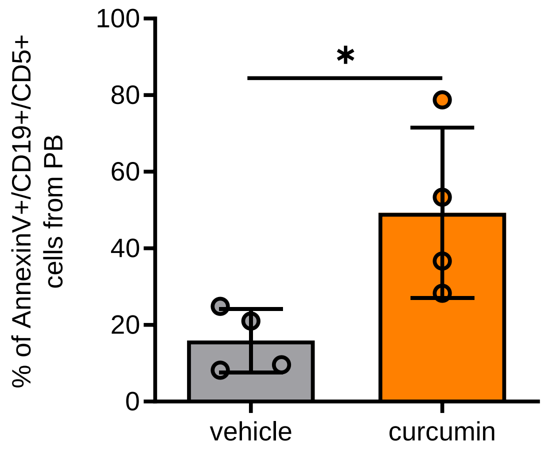

Supplementary Figure S7. Percentage of AnnexinV+/CD19+/CD5+ cells from Peripheral Blood (PB) of Eμ-TCL1 mice treated with vehicle or curcumin, after 70 days. Data are presented as mean ± SD. *P<0.05, as determined by paired t test.

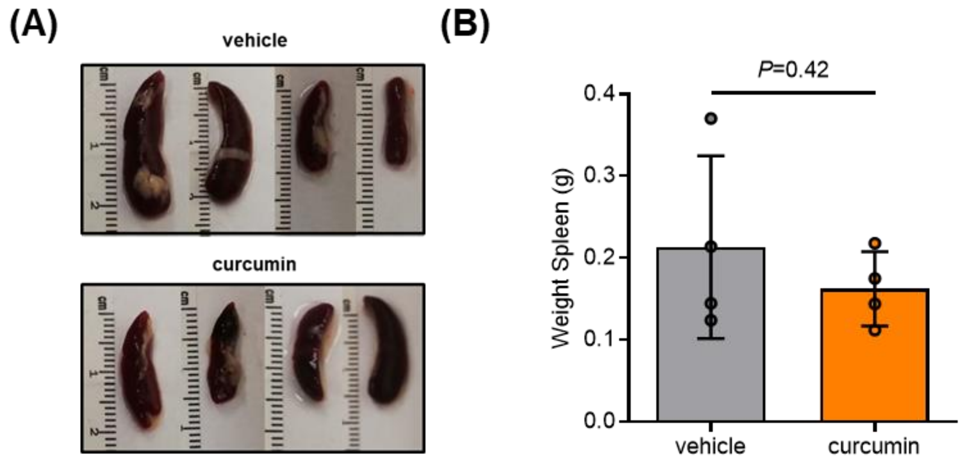

Supplementary Figure S8. Spleen dimensions of vehicle and curcumin-treated Eμ-TCL1 mice. At day 70, the animals were sacrificed and their spleens were removed and weighed. (A) Spleens of Eμ-TCL1 treated with curcumin (50mg/kg/day) or vehicle (corn oil) at day 70. (B) Bar graphs with data points of spleen weight of vehicle and curcumin-treated mice. Data are presented as mean ± SD. *P* value determined by paired t test.
